# Ru@Carbon Nanotube Composite Microsponge: Fabrication in Supercritical CO_2_ for Hydrogenation of *p*-Chloronitrobenzene

**DOI:** 10.3390/nano12030539

**Published:** 2022-02-04

**Authors:** Xianghong Ge, Hui Liu, Xingxing Ding, Yanyan Liu, Xingsheng Li, Xianli Wu, Baojun Li

**Affiliations:** 1 Zhengzhou Key Laboratory of Low-Dimensional Quantum Materials and Devices, Department of Physics, College of Science, Zhongyuan University of Technology, 41 Zhongyuan Road, Zhengzhou 450007, China; 5820@zut.edu.cn; 2 Research Center of Green Catalysis, College of Chemistry, Zhengzhou University, 100 Science Road, Zhengzhou 450001, China; lh610@zznu.edu.cn (H.L.); lyycarbon@henau.edu.cn (Y.L.); 18790251927@sina.com (X.L.); wuxianli@zzu.edu.cn (X.W.); 3 School of Chemistry and Chemical Engineering, Zhengzhou Normal University, 6 Yingcai Road, Zhengzhou 450044, China; 4 Department of Applied Chemistry, College of Science, Henan Agricultural University, 95 Wenhua Road, Zhengzhou 450002, China

**Keywords:** Ru nanoparticles, carbon nanotubes, supercritical CO_2_, catalytic hydrogenation, selective hydrogenation

## Abstract

Novel heterogeneous catalysts are needed to selectively anchor metal nanoparticles (NPs) into the internal space of carbon nanotubes (CNTs). Here, supercritical CO_2_ (SC-CO_2_) was used to fabricate the Ru@CNT composite microsponge via impregnation. Under SC-CO_2_ conditions, the highly dispersive Ru NPs, with a uniform diameter of 3 nm, were anchored exclusively into the internal space of CNTs. The CNTs are assembled into a microsponge composite. The supercritical temperature for catalyst preparation, catalytic hydrogenation temperature, and time all have a significant impact on the catalytic activity of Ru@CNTs. The best catalytic activity was obtained at 100 °C and 8.0 MPa: this gave excellent selectivity in the hydrogenation of *p*-chloronitrobenzene at 100 °C. This assembly strategy assisted by SC-CO_2_ will be promising for the fabrication of advanced carbon composite powder materials.

## 1. Introduction

Organic nanotubes open intriguing possibilities to introduce other matter into the cavities, which may lead to nanocomposite materials with interesting properties or behavior different from the bulk [1,2,3]. Carbon nanotubes (CNTs), as one type of organic nanotube, have received a great deal of attention with potential applications in energy storage and conversion, sensors, reinforcement for composites, optical and electronic devices, and support for heterogeneous catalysis [4,5,6,7,8,9,10,11,12]. As nanoreactors with a well-defined structure in terms of inner hollow cavities, the CNTs are excellent spatial carriers for the confinement effect. The ability to modify the redox properties via confinement in CNTs is expected to be of significance for many catalytic reactions [13,14,15,16,17,18,19,20]. Many advanced powder materials and other components can be designed via assembly of CNTs [21,22,23]. For example, CNTs have been used for the first time to support ruthenium NPs for the hydrogenation of *p*-chloronitrobenzene (*p*-CNB) to selectively produce *p*-chloroaniline, which is one order of magnitude higher than a commercial Ru/Al_2_O_3_ catalyst [24]. The unique properties of CNTs could lead to metal NPs with structural changes on the nanoscale, leading to dramatic changes in the catalytic properties [25,26,27]. However, the outer surface of CNTs becomes a negative factor for efficient utility of the confinement effect of catalysts. It remains difficult to introduce NPs readily and exclusively into the interior space of CNTs and avoid loading into the outer surface of CNTs. This obstacle must be overcome for future industrial production and applications. Nanoscale CNT powder catalysts often face separation problems. The assembly of CNTs is important for their use as catalysts due to the advantages of CNTs in the separation and operation issues. CNTs and the corresponding composites can exist in the powder or bulk microsponge form to provide an opportunity for the development of the CNT catalysts [28,29,30].

Impregnation methods are widely used in traditional catalyst preparation but with limited success. The high surface tension of the immersion medium prevents the catalyst components from entering into the internal surface of the CNTs. Therefore, some new strategies must be explored. Supercritical fluids (CO_2_ or other) possess zero surface tension. CO_2_ is non-toxic and environmentally benign. Supercritical CO_2_ (SC-CO_2_) fluid is very suitable for use in the impregnation of CNTs with NPs [15,31,32,33]. The SC-CO_2_ fluid-assisted method may be developed into an effective strategy for facile construction of advanced powder materials. As a highly active catalyst component for many selective hydrogenation of organics, Ru is an appropriate object for the design and fabrication of advanced heterogeneous catalysts [34,35,36]. The selective hydrogenation of *p*-chloronitrobenzene (*p*-CNB) produces *p*-chloroaniline (*p*-CAN), which is an important reaction for the synthesis of pesticides, dyes, and medicines and is also valuable as a probe reaction to evaluate the model catalysts for selective hydrogenation [37,38,39].

Here, a composite microsponge of multiwall carbon nanotubes (CNTs) was fabricated in SC-CO_2_. Ru NPs were then exclusively loaded into the internal pores of CNTs, assisted by SC-CO_2_ and methanol. The Ru@CNT microsponge was used as a catalyst for the selective hydrogenation of *p*-CNB with high catalytic activity and selectivity. The Ru@CNT microsponge is an excellent heterogeneous powder catalyst and can be separated from reaction mixtures. The SC-CO_2_ may be a superior reaction medium for high-performance catalyst preparation.

## 2. Experimental

### 2.1. Preparation of Catalysts

All of the chemicals were commercially obtained and used without further purification. RuCl_3_·*n*H_2_O (China) (0.0811 g), dissolved in CH_3_OH (China) (5 mL), and CNTs (China) (0.6000 g) were added to a stainless steel vessel (50 mL). The vessel was filled, set to 8.0 MPa and 40 °C for CR1 and 100 °C for CR2 for 1 h, and then naturally cooled to room temperature. The resulting solid was dried at 100 °C for 3 h after the CO_2_ was relieved slowly. The sample was then introduced into a self-assembly device, and the reduction reaction was performed in a hydrogen flow of 140 mL·min^−1^ at 200 °C for 2 h and then at 300 °C for 1 h. The catalysts were obtained and denoted as CRn.

### 2.2. Characterization

The morphology of the as-prepared product was studied with transmission electron microscopy (TEM, Hillsboro, OR, USA, FEI Tecnai G2 F20 S-Twin electron microscope, operating at 200 kV). The phase structure of the as-prepared product was characterized with X–ray diffraction (XRD, Hannover, Germany, Bruker D8 advance with Cu Kα λ = 1.5418 Å). Fourier transform infrared spectra (FTIR) were recorded on a Fourier Bruker Tensor-27 spectrophotometer (Bruker, Germany) with pressed KBr pellets from 400 to 4000 cm^–1^ region. The X–ray photoelectron spectrum (XPS) was recorded on a PHI Quantera SXM spectrometer (Chigasaki, Japan) with an Al Kα = 1486.60 eV excitation source, where binding energies were calibrated by referencing the C1s peak (284.8 eV) to reduce the sample charge effect. The TG−DSC measurements were performed by heating the sample on a Netzsch STA 409PC differential thermal analyzer (Selb, Germany).

### 2.3. Catalysis

The *p*-CNB (378 mg), catalyst (10 mg), magnetic stir bar, and ethanol (50 mL) were added into autoclave (150 mL), and then, the reaction system was purged with hydrogen three times to remove air. The hydrogenation reaction was performed under 2.0 MPa of H_2_ pressure at defined temperatures with stirring at 960 rpm (see the figure note for specific reaction conditions). After the specified reaction time, the reaction was stopped, and the product was centrifuged and measured with gas chromatography.

## 3. Results and Discussion

**Figure 1:**
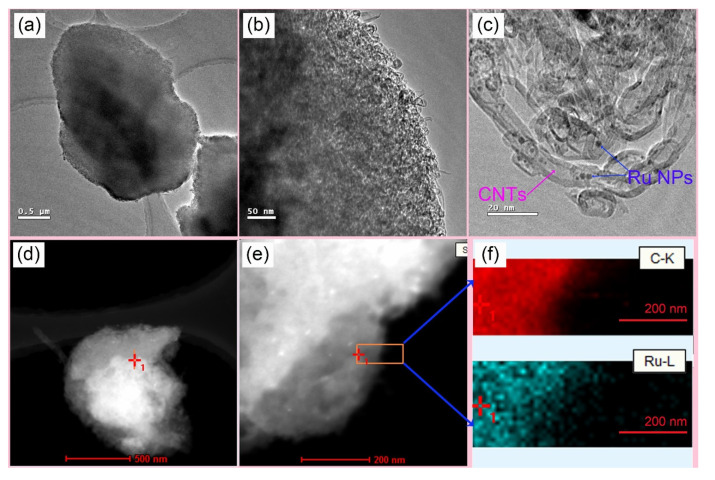
(**a**–**c**) TEM images of CR2; (**d**,**e**) high-angle annular dark field (HAADF) images of CR2; (**f**) EDS mapping of the orange rectangle region in (**e**).

The TEM images of catalysts display the typical structure and morphology of the Ru@CNT composite microsponge in Figure 1. Figure 1a–c shows the TEM images with different magnification of CR2, and Figure 1d,e shows the HAADF images of CR2. No metal particles can be seen in Figure 1a,b,d with the lower magnification. As a contrast, there are many bright dots distributed in Figure 1e, and these bright dots are Ru particles. The EDS mapping of the orange rectangle region of Figure 1e (shown in Figure 1f) indicate that the prepared samples are uniformly distributed. More distinctly, as shown in Figure 1c, the CNT microsponge particles possess sizes of micrometers. An average internal diameter of 5–10 nm was observed for CNTs. There are larger and smaller dark dots, which are the Ru particles. They are well decorated successfully in the CNTs and nearly monodisperse with no agglomeration. The average particle size of Ru NPs prepared in SC-CO_2_ is concentrated at about 3 nm (detailed calculation shown in Figure S1; see Supplementary Materials). The open pores of CNTs allow inorganic salts to enter the interior of the CNTs assisted by the SC-CO_2_ medium. This effective diffusion also ensures diffusion of reactants and products during the catalytic reaction. When SC-CO_2_ was displaced, inorganic ions were restored to their internal space. Most Ru NPs entered into CNTs and were well dispersed (Figure 1d–f). The CNTs were highly dispersed in the reaction mixture due to the unique properties of SC-CO_2_. After the SC-CO_2_ was relieved, the disorderly dispersed CNTs crossed each other, piled up, and formed a microsponge. These microparticles can be easily separated from the liquid mixture. This SC-CO_2_ immersion method showed obvious positive effects. The preparation process for the Ru@CNT composite microsponge provides a method for preparing many composite catalysts.

**Figure 2:**
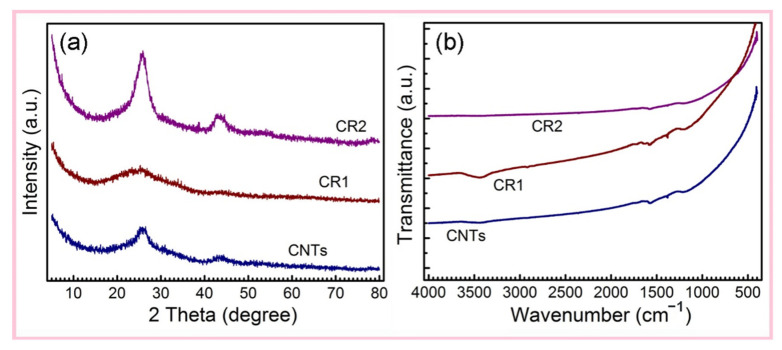
(**a**) XRD patterns and (**b**) FTIR spectra of CR1, CR2, and CNTs.

The XRD patterns of CNTs and Ru@CNTs show characteristic diffraction peaks of CNTs at 26.2° and 42.6°. Since the content of Ru in the prepared samples is very small and the size of the Ru particles is nanoscale, there are no clear diffraction peaks of Ru (Figure 2a) [40,41,42,43]. This indicates that Ru NPs are highly dispersed with no agglomeration. The FTIR spectra of CNTs and Ru NPs@CNTs have very weak characteristic CNT absorption peaks from 1600 cm^−1^ to 1450 cm^−1^; the peak nearly at 1580 cm^–1^ is assigned to a *v* (C=C) stretching vibration (Figure 2b) [7,44]. The surface groups of CNTs did not increase via the supercritical fluid impregnation and high-temperature reduction method. It suggests that the chemical structure of CNTs maintained stability under experimental conditions.

**Figure 3:**
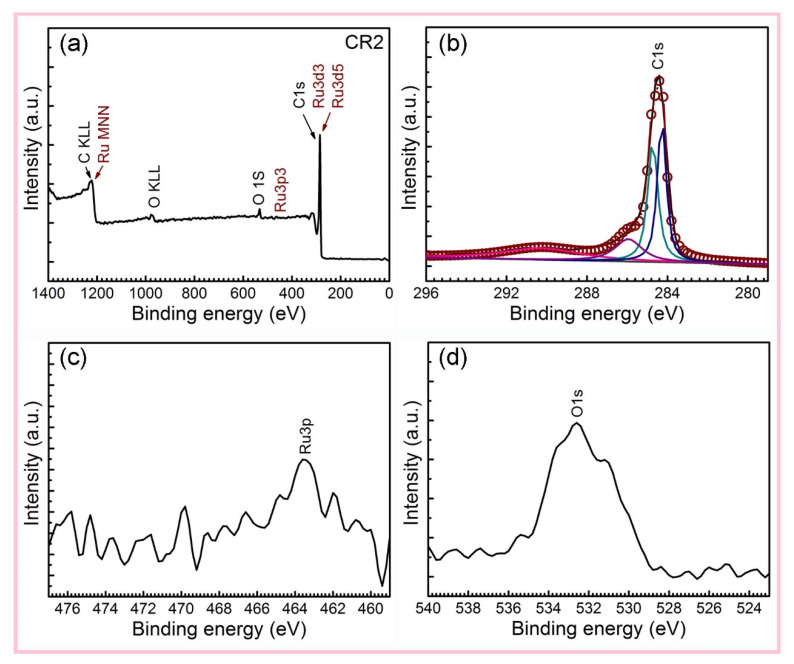
XPS spectra of (**a**) CR2, and fine structures of (**b**) C1s, (**c**) Ru3p, and (**d**) O1s spectra.

The TG−DSC analysis of CR*n* shows that the mass fractions of non-volatile impurities of CR1 and CR2 at high temperature are 8.9% and 9.0% (Figure S2; see Supplementary Materials). The loading amount of Ru in CR1 and CR2 is calculated as 4.5 wt% and 4.6 wt% from TG−DSC analysis, respectively (calculation process; see Supplementary Materials). The XPS spectra also showed covalent bonds of C, O, and Ru atoms in the composites (Figure 3 and Figure S3; see Supplementary Materials) [22]. The quality ratio of Ru elements was also concluded from the XPS spectra. The weight ratio of Ru measured by XPS suggests that the mass fraction of Ru loading on the outside of CNTs is 0.58 wt%. The XPS data further show that the internal Ru loading content for CR2 is calculated as 4.02 wt%. The ratio of Ru into the internal space is equal to 87.4%. This confirms that that the supercritical fluid deposition method is highly effective for anchoring NPs into CNTs.

**Figure 4:**
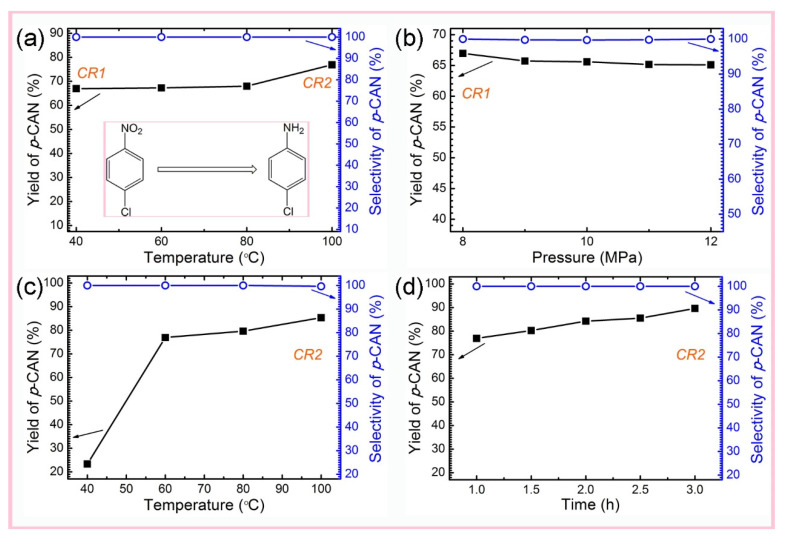
The catalytic performance in hydrogenation of *p*-CNBs with catalysts prepared (**a**) in SC-CO_2_ at various supercritical temperatures under a supercritical pressure of 8.0 MPs for 1 h, (**b**) under various supercritical pressure at 40 °C for 1 h (catalytic performance of CR2), (**c**) at various reaction temperatures under a hydrogen pressure of 2.0 MPs for 1 h, and (**d**) at 100 °C under 2.0 MPs hydrogen for various times.

The selective hydrogenation of *p*-CNB was used to evaluate the performance of the prepared catalysts and optimize the catalyst preparation parameters. The catalysts prepared at various supercritical temperatures (P = 8.0 MPa, t = 1 h) significantly influenced the yields of *p*-CAN. The optimal supercritical temperature should be 100 °C (Figure 4a). The critical conditions of supercritical fluid cannot be reached at a low temperature. At high temperatures, the absolute solubility under supercritical CO_2_ is strong, which. in turn. affects the Ru adsorption in CNTs. Meanwhile, some Ru ions or Ru NPs aggregated due to the high temperature. Catalysts prepared at different supercritical CO_2_ temperatures provided high-quality selectivity (almost 100%) of *p*-CAN (Figure 4a). The lower temperature can meet the requirements for catalyst preparation.

Figure 4b shows that upon fixing other conditions (T = 40 °C, t = 1 h), the supercritical pressure has a great influence on the activity of the corresponding catalyst. The selectivity of *p*-CAN was as high as 99.7% (Figure 4b). The catalytic activity of the catalyst prepared under the supercritical pressure of 8.0 MPa is the best one. This higher pressure can cause the relative content of the Ru active components in the supercritical fluid to decrease; thus, Ru adsorption quantity on the active carrier is negatively affected and decreased. The influence of the hydrogenation temperature on the catalytic performances was investigated with a catalyst prepared under supercritical conditions (100 °C, 8.0 MPa, 1 h). A reaction temperature of 40–60 °C has great influence on catalytic activity. The effect of temperature gradually decreased after 60 °C. The highest yield of *p*-CAN emerged at 100 °C, which is slightly higher than 60 °C (Figure 4c). The selectivities of *p*-CAN were all higher than 97%, regardless of the reaction temperature (Figure 4c). The selectivity began to reduce at 100 °C due to dechlorination reactions at a high temperature. The hydrogenation time also affected the catalytic performance (Figure 4d). The conversion of catalytic hydrogenation increased with time and reached 90% at 100 °C with the best catalyst. Due to the microsponge structure of Ru@CNT catalysts, they can be easily separated from the reaction mixtures via simple filtration and static sedimentation. Compared with other type of catalysts [24,45,46,47], the obtained catalyst fabricated in SC-CO_2_ has a better performance with high activity, shorter time, and better selectivity.

## 4. Conclusions

The SC-CO_2_ was used to fabricate the Ru@CNT composite microsponge via an impregnation method. Under the SC-CO_2_ conditions, the highly dispersive Ru NPs, with a uniform diameter of 3 nm, were exclusively anchored into the internal space of CNTs. The supercritical temperature for catalyst preparation, catalytic hydrogenation temperature, and time all have a significant impact on the catalytic activity of Ru@CNTs. The best catalytic activity for the hydrogenation of *p*-CNB can be obtained under 100 °C with a catalyst prepared at a supercritical temperature of 100 °C and a supercritical pressure of 8.0 MPa. These results indicate that SC-CO_2_ may be a useful medium for the fabrication of advanced functional materials and heterogeneous catalysts.

## Data Availability

The data presented in this study are available on request from the corresponding author.

## Supplementary Materials

The following supporting information can be downloaded at https://www.mdpi.com/article/10.3390/nano12030539/s1, Figure S1: Size of particle diameter measured: (a) Ru particles selected, (b) corresponding diameter size; Figure S2: TG and DTG curves of (a) CR1 and (b) CR2; Figure S3: Fine XPS spectra of Ru3d of CR2.
