# Supplementary material for: Ru@Carbon Nanotube Composite Microsponge: Fabrication in Supercritical CO2 for Hydrogenation of p-Chloronitrobenzene
Source: Nanomaterials (Basel). 2022 Feb 4;12(3):539. doi: 10.3390/nano12030539 (PMC8839890; doi:10.3390/nano12030539)
Supplement: Supplementary file 1 [file nanomaterials-12-00539-s001.zip › nanomaterials-1567957-supplementary.pdf]

nanomaterials

Supplementary Materials

Ru@Carbon Nanotube Composite Microsponge: Fabrication in Supercritical CO_2 for Hydrogenation of *p*-Chloronitrobenzene

Xianghong Ge [1,*], Hui Liu[2,3], Xingxing Ding [1], Yanyan Liu[2,4], Xingsheng Li [2], Xianli Wu [2] and Baojun Li[1,*]

[1] Department of Physics, College of Science, Zhongyuan University of Technology; Zhengzhou Key Laboratory of Low-dimensional Quantum Materials and Devices, 41 Zhongyuan Road, Zhengzhou 450007, China; 5820@zut.edu.cn
[2] Research Center of Green Catalysis, College of Chemistry, Zhengzhou University, 100 Science Road, Zhengzhou 450001, China; lh610@zznu.edu.cn (H.L.); lyycarbon@henau.edu.cn (Y.L.); 18790251927@sina.com (X.L.); wuxianli@zzu.edu.cn (X.W.)
[3] School of Chemistry and Chemical Engineering, Zhengzhou Normal University, 6 Yingcai Road, Zhengzhou 450044, China
[4] Department of Applied Chemistry, College of Science, Henan Agricultural University, 95 Wenhua Road, Zhengzhou 450002, China
* Correspondence: 5765@zut.edu.cn (X.G); lbjfcl@zzu.edu.cn (B.L.)

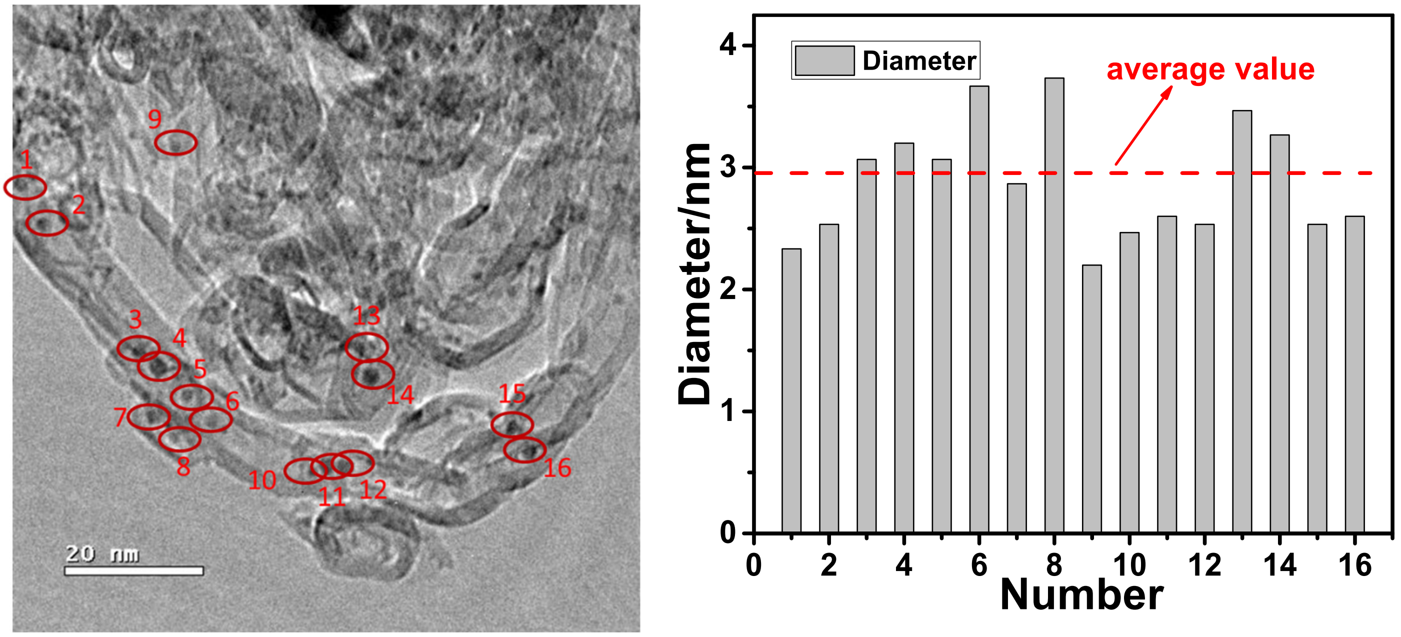

(**a**) (**b**)

Figure S1. Size of particle diameter measured: (**a**) Ru particles selected, (**b**) corresponding diameter size.

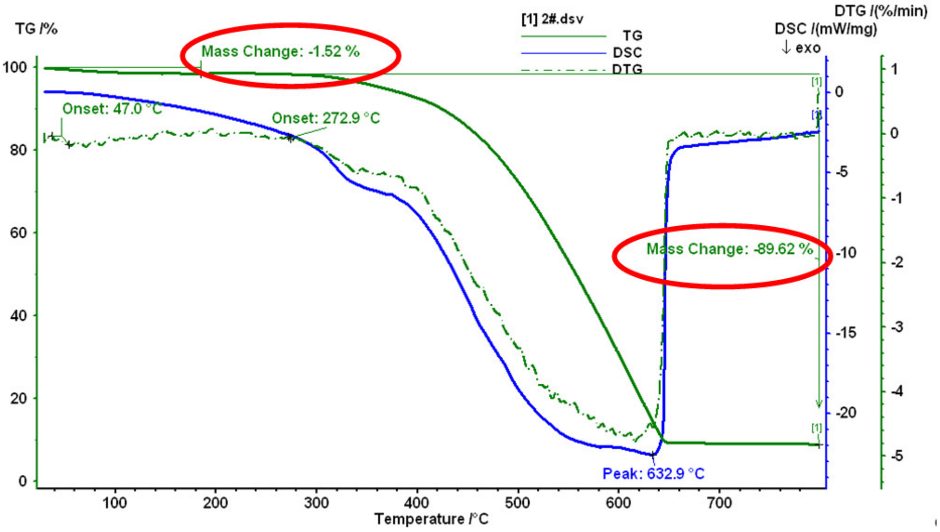

(**a**)

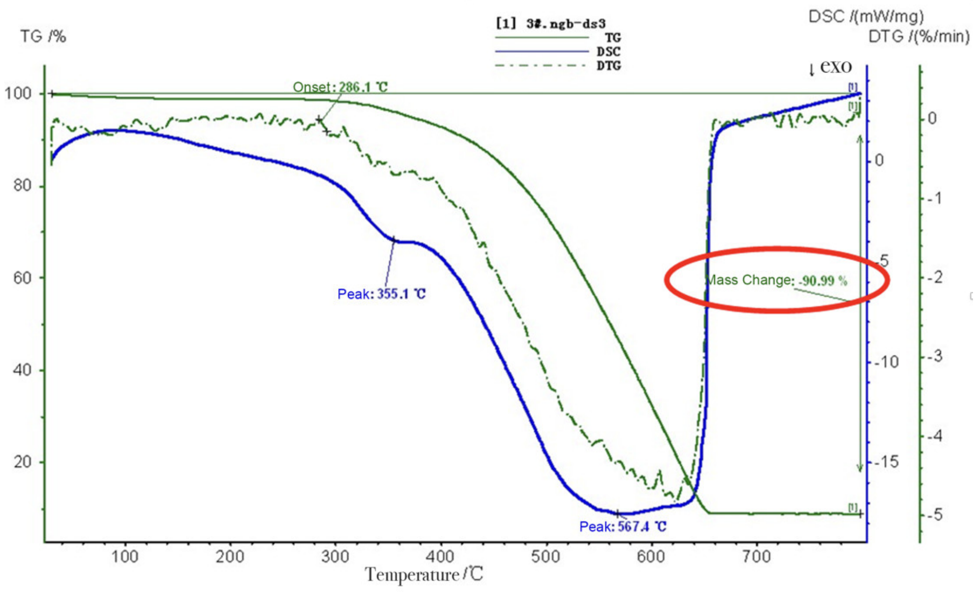

(**b**)

Figure S2. TG and DTG curves of (**a**) CR1 and (**b**) CR2.

The purity of CNTs provided by the manufacturer is about 95%, and grayscale of ash of CNTs in the total composition is 5% * 0.6 / (0.6 + 0.0811) = 4.4%, where „0.6" and „0.0811" represent the mass of CNTs and $RuCl_3 \cdot nH_2O$, respectively. According to the mass change, the ruthenium of load capacity in CR1 and CR2 is 8.9% − 4.4% = 4.5% and 9.0% − 4.4% = 4.6%, respectively. The quality ratio of ruthenium is only 4.6 wt%, the mass fraction of ruthenium loading on the outside of CNT is 0.58 wt%. The loading content of internal ruthenium of CR2 is: 4.6% − 0.58% = 4.02%, and the ruthenium into the internal accounts for 4.02%/4.6%=87.4%.

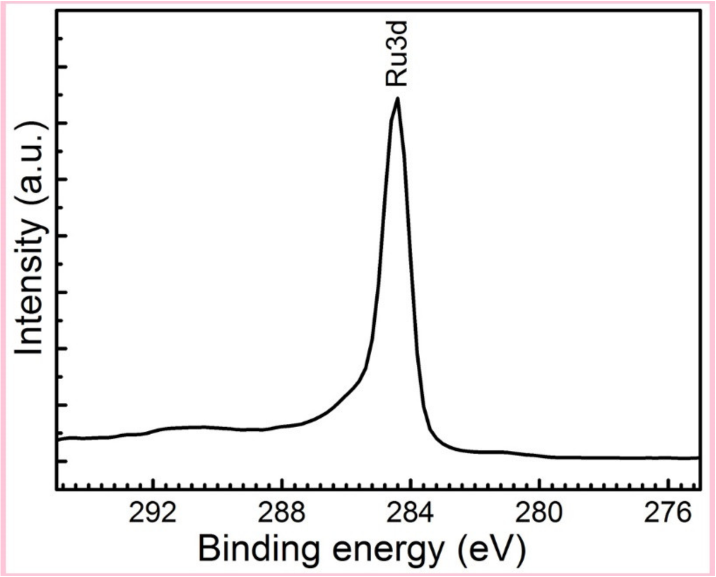

Figure S3. Fine XPS spectra of Ru3d of CR2.
